# Metabolomics Elucidates Dose-Dependent Molecular Beneficial Effects of Hesperidin Supplementation in Rats Fed an Obesogenic Diet

**DOI:** 10.3390/antiox9010079

**Published:** 2020-01-16

**Authors:** Maria Guirro, Andreu Gual-Grau, Albert Gibert-Ramos, Juan Maria Alcaide-Hidalgo, Núria Canela, Lluís Arola, Jordi Mayneris-Perxachs

**Affiliations:** 1 Eurecat, Centre Tecnològic de Catalunya, Technological Unit of Omics Sciences, 43204 Reus, Spain; maria.guirro@eurecat.org (M.G.); nuria.canela@eurecat.org (N.C.); 2 Nutrigenomics Research Group, Department of Biochemistry and Biotechnology, Universitat Rovira i Virgili. Marcel.lí Domingo, 1, 43007 Tarragona, Spain; andreu.gual@urv.cat (A.G.-G.); Albert.gibert@urv.cat (A.G.-R.); 3 Eurecat, Centre Tecnològic de Catalunya, Technological Unit of Nutrition and Health, 43204 Reus, Spain; juanmaria.alcaide@eurecat.org; 4 Department of Diabetes, Endocrinology and Nutrition, Dr. Josep Trueta University Hospital, and Girona Biomedical Research Institute (IDIBGI), 17007 Girona, Spain; 5 Center for Physiopathology of Obesity and Nutrition (CIBERobn), Instituto de Salud Carlos III, 28029 Madrid, Spain

**Keywords:** metabolic syndrome, cafeteria diet, polyphenols, citrus flavanones, hesperidin, metabolomics, metabolic phenotyping, gut microbiome

## Abstract

Metabolic syndrome (MetS) is a global epidemic concern. Polyphenols are proposed as good candidates for its prevention, although their mechanisms are not fully understood. The gut microbiota seems to play a key role in polyphenol beneficial effects. Here, we assessed the effects of the citrus polyphenol hesperidin combining an untargeted metabolomics approach, which has an inherent potential to elucidate the host-microbiome interplay, with extensive anthropometric and biochemical characterizations and integrating metabolomics results with our previous 16S rRNA bacterial sequencing data. The rats were fed either a standard or an obesogenic cafeteria diet (CAF) for 17 weeks. After nine weeks, rats were supplemented with vehicle; low- (H1), or high- (H2) hesperidin doses. CAF animals developed MetS features. Hesperidin supplementation in CAF rats decreased the total cholesterol, LDL-C, and free fatty acids. The highest hesperidin dose also ameliorated blood pressure, insulin sensitivity, and decreased markers of arterial stiffness and inflammation. Metabolomics revealed an improvement of the lipidomic profile, decreases in circulating amino acids, and lower excretions of inflammation- and oxidative stress-related metabolites. *Bacteroidaceae* increases in the CAF-H2 group paralleled higher excretions of microbial-derived metabolites. Overall, our results provide detailed insights into the molecular effects of hesperidin on MetS and suggest that it is a promising prebiotic for the treatment of MetS and related conditions.

## 1. Introduction

Metabolic syndrome (MetS) is defined as a clustering of interrelated cardio-metabolic risk factors, including abdominal obesity, insulin resistance (IR), hypertension, and dyslipidaemia, which increases the risk of developing both cardiovascular disease (CVD) and type 2 diabetes (T2D) [1,2,3]. The MetS is highly prevalent worldwide (about 20–30% of all adults), and it is increasing in relation to the increasing incidence of obesity and T2D. Therefore, it has become a major public health concern and the seek for effective treatments is crucial [4]. The current guidelines point out therapeutic lifestyle modification, including reducing body weight and increasing physical activity, as the first step in the management of MetS [1,5]. However, a consensus is lacking regarding the optimal diet [6]. Despite this, epidemiological evidence highlights the importance of the consumption of fruits and vegetables. In particular, plant polyphenols have emerged as potential effective nutritional strategies for improving the health of patients with MetS [7,8]. Among the wide variety of dietary polyphenols, hesperidin, which is a flavanone glycoside found abundantly in citrus fruits, has shown a promising role against CVD due to their antihypertensive, lipid-lowering, hypoglycemic, antioxidant, and anti-inflammatory properties [9,10,11].

Despite this, the mechanisms by which hesperidin induces these benefits are far from being fully understood. In fact, the few intervention studies that have been performed to date with pure hesperidin indicate a possible role of hesperidin in improving endothelial function, whereas the antihypertensive, lipid-lowering, antioxidant, and anti-inflammatory effects are inconsistent [12,13,14,15]. However, most of these studies have assessed the hesperidin effects while using classical single biomarkers. Taking that polyphenols have multiple biochemical targets and physiological actions, and that most chronic diseases are multifactorial, into account, these markers provide a reductionist and incomplete picture. Conversely, metabolomics, which involves the comprehensive study of complete profiles of small molecules in response to stimuli, provides a holistic picture, and holds great potential to tackle the complex relationship between nutrition and health.

Importantly, the vast majority of polyphenols reach the colon intact, where they are extensively metabolized by the gut microbiota into several lower molecular weight catabolites, which might be responsible for the beneficial health effects, rather than the original compounds [16,17]. Many of these catabolites are absorbed in the colon and appear in blood and urine, merging with endogenous metabolites, thereby altering the metabolome and influencing host health. In turn, polyphenols can modulate gut microbial composition or functionality, which affects the release of microbial-derived metabolites [18]. Because of this, metabolomics has an inherent potential for elucidating the host-microbiome interplay and might contribute to a better understanding of the underlying mechanisms that are involved in polyphenol-derived health effects [17,19].

A CAF diet in rodents has been shown to be a robust model of human MetS [20]. We have previously shown that hesperidin supplementation in CAF-fed rats altered microbial diversity and functionality at the proteome level [21]. Here, we hypothesize that hesperidin supplementation will exert protective effects in rats with MetS. We report, for the first time, a comprehensive untargeted metabolomic approach combined with an extensive anthropometric and biochemical characterization and integration with our previous bacterial data to study the effects and underlying mechanisms of different doses of hesperidin on rats fed a CAF diet. To this end, we measured the metabolic profiles of blood and urines samples by ^1^H nuclear magnetic resonance (NMR) and several markers of glucose and lipid metabolism, inflammation, endothelial function, arterial stiffness, and blood pressure.

## 2. Materials and Methods

### 2.1. Hesperidin Source

Hesperidin was generously provided by NUTRAFUR S.A. (Murcia, Spain). Hesperidin was extracted from the fruit peel of *Citrus sinensis* (sweet orange) and the purity was 93% (HPLC). Hesperidin has a chiral carbon atom at position 2 and, therefore, exists as two isomeric forms: 2S and 2R hesperidin. In naturally unprocessed citrus fruits, hesperidin is almost all 2S epimer [22], and in fresh sweet orange juice the 2S isoform constitutes at least 92% of hesperidin [23], being the most bioactive isoform [24]. However, during industrial processing, the 2S epimer is transformed to the 2R isomer, and commercial hesperidin usually contains a ratio 2S:2R that is close to 1.5:1. In our hesperidin extract, the proportion of 2S hesperidin was precisely 66%, as determined by NMR (Figure S1).

### 2.2. Animals, Diets and Treatments

Forty-eight eight-week-old male Sprague-Dawley rats (Charles River Laboratories, Barcelona) (average weight 251 ± 2.5 g) were used. The rats were individually housed at 22 °C with a 12h period of light/dark and had free access to water and food. After a quarantine period of one week, the rats were randomly split into two dietary groups (*n* = 24): STD group, fed ab libitum with a standard chow (Teklad global 18% protein, Envigo) for nine weeks; CAF group, fed with a cafeteria (CAF) diet for nine weeks (Figure S2). The CAF diet included the following components: standard chow, bacon (8–12g), biscuits with pâté (12–15 g), biscuits with cream cheese (10–12 g), carrot (6–9 g), muffins (4–5 g), and 100 mL of milk with sugar (220 g/L). The STD diet (3.1 kcal/g) contained 24% calories from protein, 18% from fat, and 58% from carbohydrates, whereas the caloric distribution of the CAF diet was 10% protein, 41% fat, and 49% carbohydrates.

Blood pressure and body composition were measured at the beginning of the 10th week to determine the development of the MetS characteristics in rats fed a CAF diet. Blood samples from the saphenous vein were also obtained after 8 h of diurnal fasting. Thereupon, each dietary group was divided into three treatment groups (*n* = 8): vehicle (V), low dose hesperidin (H1), or high dose hesperidin (H2). Therefore, two groups in each dietary regime were supplemented every day (at 9:00 h) with hesperidin that was dissolved in low-fat condensed milk diluted 1:1 at a dose of 40 mg/kg (STD-H1 and CAF-H1) or 100 mg/kg (STD-H2 and CAF-H2) for eight weeks. The STD-V and CAF-V groups received the corresponding volume of low-fat condensed milk. The treatments were orally administered while using a 1mL syringe. While considering, on average, a rat weight of 450 g, the doses of hesperidin used were equivalent to the daily consumption of 500 mg and 1350 mg of hesperidin for a 60 kg human, respectively [25]. The lower dose was selected on the basis of hesperidin effectiveness and achievable dietary intakes [12,13,26]. Thus, 500 mg of our commercial hesperidin extract are equivalent to 330 mg of the most-active 2S hesperidin isoform, an amount that can be found in 500 mL of fresh orange juice [27], which has shown vascular protective effects [13]. In addition, 500 mg of commercial hesperidin capsules have shown beneficial effects on vascular function, oxidative stress, and inflammation in patients with the MetS and T2D [12,26]. The higher dose was based on the beneficial effects of hesperidin on MetS-related alterations in rodent models of diet induced obesity [28,29], and it could be used in the designing functional foods. 

At the beginning of the 18th week, the blood samples were obtained by saphenous vein puncture as described above. Rats were also placed in individual metabolic cages so as to collect 24-h urinary samples. The urine samples collected in a solution of 1% (wt/vol) sodium azide, were filtered and kept at −80 ℃ until analysis. One week later, rats were fasted for 8h and anaesthetized with pentobarbital sodium (80 mg/kg body weight), blood was collected through cardiac puncture, and serum was prepared by centrifugation. Several tissues (brain, cecum, heart, kidney, liver, soleus and gastrocnemius muscles, and white adipose depots (epididymal, inguinal, mesenteric, retroperitoneal)), were rapidly removed, weighted, and frozen in liquid nitrogen. All of the samples were stored at −80 ℃ until analysis.

The study was conducted in accordance with the European Communities Council Directive (86/609/EEC) and the Animal Ethics Review Committee for Animal Experimentation of the University Rovira i Virgili (Tarragona, Spain) and by Department of Territory and Sustainability of the Generalitat de Catalunya (Catalan Government permission number 10061) approved it.

### 2.3. Blood Pressure Measurement

Systolic blood pressure (SBP) measurements were performed once a week during the treatment period (from the 10th to the 18th week) while using the tail-cuff method [30]. Before each measurement, the rats were kept at 32 ℃ for 15 min. to calm the animals and to make the pulsations of the tail artery detectable. The SBP measurements were performed at the same time of the day (between 15:00 and 17:00) while using the LE5001 blood pressure meter (Harvard apparatus, Barcelona, Spain) and SBP values were obtained as the average reading of at least five measurements. All of the measurements were taken in a peaceful environment and by the same person for each animal throughout the study to minimize stress-induced variations in SBP. Before starting the SBP measurements, we established a two-week training period (weeks 8th–9th) to familiarize the rats with the procedure, during which SBP was measured every day.

### 2.4. Body Composition and Adiposity Index

The EchoMRI-700™ self-contained quantitative nuclear magnetic resonance system (Echo Medical Systems, L.L.C., Houston, TX, USA) was used to determine the rats body composition (lean mass, fat mass, and total body water) without the need of anesthesia. The adiposity index (AI) was calculated as the sum of the four white adipose tissue depots weights and expressed as a percentage of the total body weight.

### 2.5. Biochemical Analyses

The levels of glucose, triacylglycerols (TG), and total cholesterol (QCA, Barcelona, Spain), and non-esterified free fatty acids (FFAs) (WAKO, Neuss, Germany) were determined in serum samples that were prepared from saphenous vein blood before and after the treatment period (10th and 18th weeks) by enzymatic colorimetric kits. The circulating levels of LDL/VLDL-C and HDL-C and those of insulin were measured from the same samples at the end of the treatment by enzymatic colorimetric kits (Bioassay systems, California, CA, USA) and while using a rat/mouse ELISA kit (Millipore, Barcelona, Spain), respectively. Insulin resistance and sensitivity were assessed while using the HOMA-IR and R-QUICKI indices with the following formulas: (Glucose x Insulin)/22.5 and 1/[log insulin (µU/mL) + log glucose (mg/dL) + log FFA (mmol/l)], respectively.

From serum samples that were obtained from cardiac puncture at the end of the study, we measured vascular cell and intercellular adhesion molecule 1 (VCAM-1 and ICAM-1) and serum monocyte chemoattractant protein 1 (MCP-1) while using rat ELISA kits (Thermo Scientific, Illinois, USA). The Neuraminidase (NA) activity was measured with the Amplex^TM^ Red Neuraminidase Assay Kit (Thermo Scientific, Illinois, IL, USA).

### 2.6. ^1^H nuclear Magnetic Resonance Spectroscopy-Based Metabolic Profiling

Serum (from saphenous vein) and urine samples at the end of the study were subjected to ^1^H NMR metabolomics analysis.

*Sample preparation*. All of the urine samples were prepared by combining 400 µL urine with 200 µL phosphate buffer (pH 7.4; 100% D_2_O) that contained 1 mM of the internal standard/L, 3-trimethylsilyl-1-[2,2,3,3-2H4] propionate (TSP), and 2 mM sodium azide. The samples were vortexed, centrifuged for 10 min. at 10,000× *g*, and then transferred to a 5 mm NMR tube.

For serum extraction, 200 µL of serum were lyophilized for 16h. The resulting pellet was resuspended by adding 1200 µL of methanol (CH3OH), 400 µL of miliQ water, and 400 µL of chloroform (CHCl_3_). The sample was vigorously vortexed for 1 min. and the sonicated for 20 min. before adding 2 mL of CHCl_3_ and 1 mL of miliQ water. The sample was vortexed again and centrifuged at 2400× *g* for 20 min. at 4 ℃. The upper layer (CH_3_OH/water) containing the serum aqueous extract and the lower organic phase (CHCl_3_/CH_3_OH) containing the lipophilic extract were separately recovered and evaporated to dryness under N_2_. The aqueous extract was reconstituted with 600 µL phosphate buffer (pH 7.4; 100% D_2_O) that contained 1 mM TSP and 2mM sodium azide, whereas the lipid extract was dissolved into 600 µL 0.01% tetramethylsilane (TMS) solution of 3:1 CDCl_3_:CD_3_OD.

The NMR spectra were measured at a 600.20 MHz frequency while using an Avance III-600 Bruker spectrometer equipped with a 5 mm PABBO BB-^1^H/D Z-GRD probe. For urine samples, a standard one-dimensional (1D) NOESY presaturation pulse sequence (RD-90°-t1-90°-tm-90°-acquire, noesypr1d) was used with water suppression. A recycle delay (RD) of 5.0 s, a mixing time (tm) of 100 ms, and an acquisition time of 3.4 s, and a 90° pulse of 21.16 µs, were used for all of the samples. Four dummy scans were used to establish spin equilibrium, and then 128 scans were collected into 64K data points with a spectral width of 16 ppm. 

For serum aqueous extracts, a standard 1D NOESY presaturation pulse sequence (noesypr1d) was used. A RD of 5.0 s, a mixing time of 100 ms, an acquisition time of 3.4 s, and a 90° pulse of 10.02 µs, were used. For each aqueous extract, four dummy scans were followed by 256 scans and collected in 64K data points with a spectral with of 16 ppm. In the case of lipophilic extracts, a 90° pulse with presaturation sequence (zgpr) was used. A RD of 5.0 s, a mixing time of 100 ms, an acquisition time of 2.94 s, and a 90° pulse of 9.92 µs, were used. After four dummy scans, a total of 256 scans were collected into 64K data points with a spectral with of 18.6 ppm.

*NMR data processing of urine and serum*. NMR spectra were processed using the software TopSpin 3.5pl4 (Bruker Biospin, UK). An exponential line broadening of 0.3 Hz was applied before Fourier transform. Spectra were manually phased, baseline corrected and referenced to the chemical shift of TSP (0.0 ppm). ^1^H NMR spectra (δ = −1.0–10.0) were digitized into consecutive integrated spectral regions with a resolution of 0.00034 ppm and 0.00025 ppm for urine and serum, respectively. In urine spectra, the water region between δ = 4.70 and 5.17 was removed to minimize that baseline effects that were caused by imperfect water suppression. The urea region was also removed (δ = 5.60–6.0). In serum aqueous extracts, the regions between δ = 4.70 and 6.0 were removed, whereas, in lipophilic extracts, the water (δ = 4.44–5.20) region and the CH_3_OH (δ = 3.26–3.44) and CHCl_3_ (δ = 7.4–7.6) regions were removed. All of the serum and urine NMR spectra were aligned by the recursive segment-wise peak alignment (RSPA) approach [31], and urine spectra were normalized with a probabilistic quotient method [32].

*Metabolite identification*. Metabolite identification was carried out while using information from the literature and public databases (Chenomx NMR Suite, Human Metabolite DataBase, Biological Magnetic Resonance Data Bank). Two-dimensional (2D) NMR experiments (COSY, TOCSY, HSQC) were acquired for a number of samples to assist or confirm metabolite identification.

### 2.7. Univariate Statistical Analysis

The outliers were discarded for subsequent analyses based on the results of a Grubbs’ test. The Kolmogorov-Smirnov and Levene’s tests assessed the normality and homoscedasticity, respectively. Data were log10-transformed when one or both of these conditions were not met. Differences between STD and CAF groups just before the treatment period (week 10) were assessed by a Student’s *t* test, whereas the group differences at the end of the treatment (week 18) were assessed while using a general linear mixed model, including diet (*D*) and treatment (*T*) and their interaction (*DxT*) as fixed factors, followed by the Tukey’s post-hoc test. For parameters measured at multiple times (such as SBP) a linear mixed model followed by the Sidak post-hoc test was used. Fixed effects included diet (*D*), treatment (*T*), time (*t*), and their corresponding interactions. The model included an unstructured covariance matrix and the subjects as a random factor. Slice tests were conducted in the presence of significant interactions. The data are presented as means ± SEM (*n* = 8) and significance was set at *p* < 0.05. The IBM SPSS statistical software package version 25.0 (SPSS, IBM Corp. Armonk, New York, NY, USA) was used for statistical analyses. 

### 2.8. Multivariate Statistical Analysis

Multivariate modelling was performed in MATLAB with the use of in-house scripts. Initially, principal component analysis (PCA) of the NMR spectra was performed while using pareto scaling to visualize patterns and outliers within the data set. This was followed by orthogonal projection to latent structures discriminant analysis (OPLS-DA). This approach was used for pair-wise comparisons between the study groups to identify discriminatory metabolites between the groups. OPLS-DA model loadings were back-transformed by multiplying all values by their standard deviation (covariance) and color plotted by their model weights (R^2^), which in the case of two classes, represent the correlation between the NMR variables (X) and the class-membership (Y). Important variables for between class discrimination are highlighted by the correlation color scale, with red indicating high significance. We used a seven-fold cross-validation approach to obtain the predictive performance of the models (Q^2^Y) and the model significance was calculated based on permutation tests with 1000 permutations.

*Clustering analysis*. Unsupervised hierarchical clustering analysis (HCA) was performed to identify the general patterns of metabolic variation between groups. Metabolites that were identified as contributing to the separation between groups through the OPLS-DA models were used for sample clustering. For a comparative analysis across different metabolites, before clustering, each metabolite concentration was standardized across samples as a *z*-score. HCA was performed while using Euclidean distances and the Ward linking method. 

*Correlation analysis*. Spearman’s correlation analysis between significant metabolites and OTUs was performed to explore the functional associations between metabolic perturbation after hesperidin supplementation and gut microbial changes (analysed previously by 16S rRNA sequencing in another paper [21]).

## 3. Results

### 3.1. A CAF Diet Induced the MetS

As expected, after nine weeks of diet, CAF-fed animals had already developed a MetS phenotype (Table 1), being characterized by increased body weight and fat mass, hypertriglyceridemia, hyperglycaemia, and elevated SBP. They also had higher levels of TC and FFA and lower relative lean mass (Table 1). These deleterious effects could be attributed to the increased energy intake and decreased cumulative protein intake that were observed in these animals when compared to STD-fed rats. At the end of the study (week 18^th^), CAF-fed animals had the same alterations in the previous parameters. They also had a higher adiposity (*p* < 0.001), elevated insulin concentrations (*p* < 0.001), lower insulin sensitivity (*p* < 0.001), and insulin resistance (*p* < 0.001). Finally, CAF feeding also resulted in increased levels in markers of endothelial dysfunction (ICAM-1, *p* = 0.041), arterial stiffness (Neuraminidase, *p* < 0.001), and inflammation (MCP-1, *p* < 0.001).

### 3.2. Hesperidin Supplementation had no Effect on Body Composition

**Figure 1:**
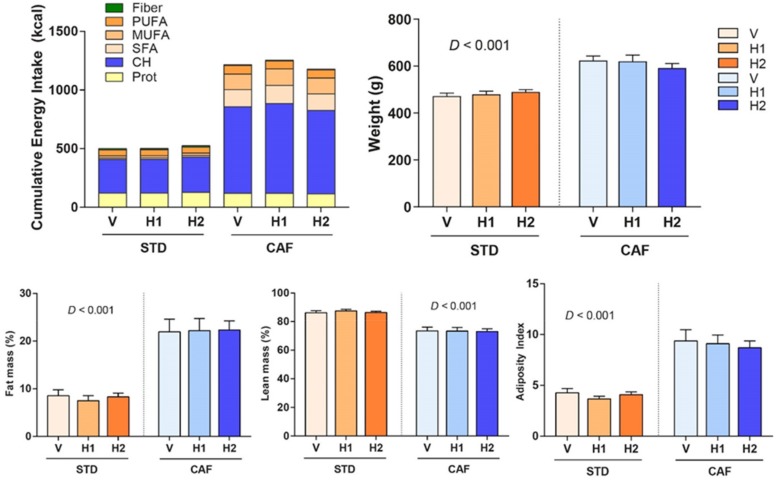
End point (week 18) cumulative energy intake, weight, fat mass, lean mass, and adiposity index of rats that were fed with a standard (STD) or a cafeteria (CAF) diet and received a daily oral dose of vehicle (V), hesperidin at 40 mg/kg (H1), or hesperidin at 100 mg/kg (H2), for the last eight weeks. Data are given as means ± SEM. CH, carbohydrates; *D*, Diet effect; MUFA, monounsaturated fatty acids; Prot, proteins; PUFA, polyunsaturated fatty acids; SFA, saturated fatty acids.

Hesperidin supplementation could not prevent weight gain in CAF-fed rats. Therefore, no significant changes in body weight, fat and lean mass percentages, and adiposity index were observed after hesperidin supplementation in CAF-fed rats when compared to those supplemented with the vehicle (Figure 1). Similar results were observed in STD-fed rats (Figure 1).

### 3.3. Hesperidin Supplementation Improved the Lipid Profile in a Dose-Dependent Manner

**Figure 2:**
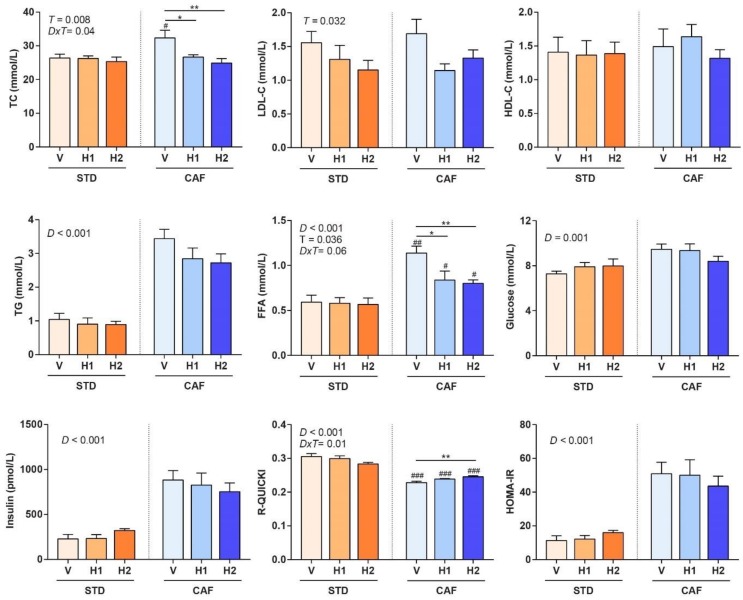
End point (week 18) lipid and glucose metabolism biochemical parameters of rats that were fed with a standard (STD) or a cafeteria (CAF) diet and received a daily oral dose of vehicle (V), hesperidin at 40 mg/kg (H1), or hesperidin at 100 mg/kg (H2), for the last 8 weeks. Data are given as means ± SEM. *D*: Diet effect; *DxT*: diet-treatment interaction; *T*: treatment effect. # *p* < 0.05, ## *p* < 0.01, ### *p* < 0.001 *vs* the corresponding supplementation group in the STD diet model. * *p* < 0.05, ** *p* < 0.01 vs the V group in the same dietary model. FFA, free fatty acids; HOMA-IR, Homeostatic model assessment of insulin resistance; R-QUICKI, revised quantitative insulin sensitivity check index; TC, total cholesterol.

Hesperidin supplementation in CAF-fed rats resulted in healthier lipid profiles in a dose-dependent manner. Therefore, the CAF-H1 and CAF-H2 rats had lower TC and LDL-C levels when compared to CAF-V rats, although no differences were observed in the HDL-C concentrations (Figure 2). The same trend was observed in the LDL-C levels of STD-fed rats. Hence, hesperidin supplementation in both dietary regimens improved the lipid profile. In addition, hesperidin supplementation decreased the circulating levels of FFA in CAF-fed rats, but not in STD-fed rats. No changes in the TG concentrations were observed after hesperidin supplementation in either STD- or CAF-fed animals as compared to their respective controls.

### 3.4. Hesperidin Supplementation Improved Response to Insulin in CAF-Fed Rats

The CAF-H2 rats showed higher insulin sensitivity, as measured by the R-QUICKI index, as compared to CAF-V rats (Figure 2), which is line with the decrease in FFA observed in these animals due to their relationship with insulin resistance. No significant changes were observed in glucose, insulin levels, or HOMA-IR in CAF-fed rats that were supplemented with hesperidin when compared to their respective controls. Hesperidin supplementation had no effect on any of the glucose metabolism parameters (glucose, insulin, R-QUICKI, HOMA-IR) in STD-fed rats.

### 3.5. The Highest dose of Hesperidin Improved SBP Following a CAF Diet

**Figure 3:**
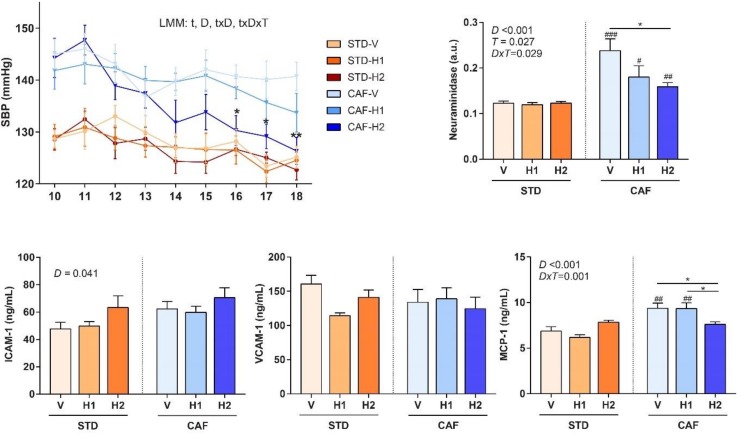
Systolic blood pressure (SBP) and end point (week 18) endothelial function parameters of rats that were fed with a standard (STD) or a cafeteria (CAF) diet and received a daily oral dose of vehicle (V), hesperidin at 40 mg/kg (H1), or hesperidin at 100 mg/kg (H2), for the last 8 weeks. Data are given as means ± SEM. *D*, Diet effect; *DxT*, diet-treatment interaction; ICAM-1, Intercellular Adhesion Molecule 1; LMM, linear mixed model; MCP-1, Monocyte Chemoattractant Protein 1; *T*, treatment effect; *t,* time effect; VCAM-1, Vascular Cell Adhesion Molecule 1. # *p* < 0.05, ## *p* < 0.01, ### *p* < 0.001 vs the corresponding supplementation group in the STD diet model. * *p* < 0.05, ** *p* < 0.01 *vs* the V group in the same dietary model.

A linear mixed model analysis of SBP showed significant *time* (*p* < 0.001) and *diet* effects (*p* < 0.001), and significant *time x diet* (*p* = 0.004) and *time x treatment x diet* (*p* = 0.05) interactions (Figure 3). Therefore, slice tests were conducted for each time and diet. They showed that the highest dose of hesperidin (H2) in CAF-fed rats reduced SBP when compared to CAF-V rats after six (130.3 ± 2.3 vs 140.7 ± 2.4, *p* = 0.010), seven (129.1 ± 2.7 vs 140.1 ± 2.9, *p* = 0.026), and eight (126.3 ± 2.6 vs 140.7 ± 2.7, *p* = 0.001) weeks of supplementation. Although, the H1 did not show significant differences when compared the V, there was a clear trend towards reducing SBP, and it is likely that a longer supplementation period could have achieved significant results.

### 3.6. Dose-Dependent Improvement in Inflammation and Markers of Arterial Stiffness after Hesperidin Supplementation in CAF-Fed Animals

Hesperidin supplementation considerably reduced the circulating levels of Neuraminidase, which is a marker of arterial stiffness, in CAF-fed rats in a dose-dependent manner (Figure 3). In addition, CAF-H2 rats had lower levels of MCP-1, an inflammation marker, when compared to CAF-H1 and CAF-V rats. After eight weeks of supplementation, no significant differences were observed in the circulating concentrations of markers of endothelial dysfunction (ICAM-1 and VCAM-1) among the groups in any dietary regime. 

### 3.7. Hesperidin Supplementation Improved the Metabolic Profiles of CAF-Fed Rats in a Dose-Dependent Manner

Pairwise OPLS-DA models were built to compare the metabolic profiles of rats that were fed either a STD or a CAF diet and supplemented with the vehicle. Significant models with good predictive abilities were obtained for the comparison of the urinary metabolic profiles (Q^2^Y = 0.81, *p* < 0.001), the serum aqueous metabolic profiles (Q^2^Y = 0.58, *p* < 0.001), and the serum lipid metabolic profiles (Q^2^Y = 0.81, *p* < 0.001). The metabolic alterations that were induced by a CAF diet as compared with a STD diet are shown in the OPLS coefficients plot for this model (Figure S3). Following a CAF-V diet, the urinary excretions of citrate, glycerophosphocholine (GPC), sucrose, *N*-methyl-4-pyridone-3-carboxamide (4-PY), and metabolites related to inflammation and oxidative stress (*N*-acetylglycoproteins [NAG], 2’-deoxycitidine) were increased when compared to a STD-V diet. However, gut microbial-host co-metabolites (Hippurate, phenylacetylglycine [PAG], 2-phenylacetamide), branched chain amino acid (BCAA) catabolism intermediates (2-oxoisocaproate [2-OIC]), taurine, and *N*-methylnicotinic acid (NMNA) were excreted in lower amounts. This lower urinary excretion of BCAA catabolites was in agreement with the higher serum levels of BCAA (valine, leucine, isoleucine) and branched chain keto acids (BCKA) (3-methyl-2-oxovalerate [2-MOV]) that were observed in CAF-V when compared to STD-V rats. Serum levels of other amino acids (proline, threonine), TCA cycle intermediates (citrate, succinate), as well as acetate, creatine, carnitine, GPC, glyceraldehyde-3-phosphate (G3P), and glucose, were also higher in CAF-V, although they had lower lactate levels. As expected, the most significant differences were found in the levels of lipids, which were increased in CAF-V when compared to the STD-V rats. The former rats had higher levels of TC, free cholesterol (FC), esterified cholesterol (EC), FFA, diglycerides (DG), TG, phospholipids (PL), phosphocholines (PC), MUFA, PUFA, linoleic acid (LA), alpha linolenic acid (ALA), EPA, and DHA.

Hesperidin supplementation in STD-fed rats only induced significant changes in the urinary metabolic profiles when compared to the vehicle (Table S1, Figures S4 and S5). The most significant difference was a much higher excretion of Hippurate and dimethyl sulfone (DMSO2) by H1 or H2. In addition, STD-H2 rats excreted lower amounts of inflammatory metabolites (NAG, fucose), acetamide, 2-OIC, and creatinine, but higher amounts of 3-hydroxyphenylpropionic acid (3-HPPA) and 3-hydroxycinammic acid (3-HCA) when compared to the STD-V rats. 

**Figure 4:**
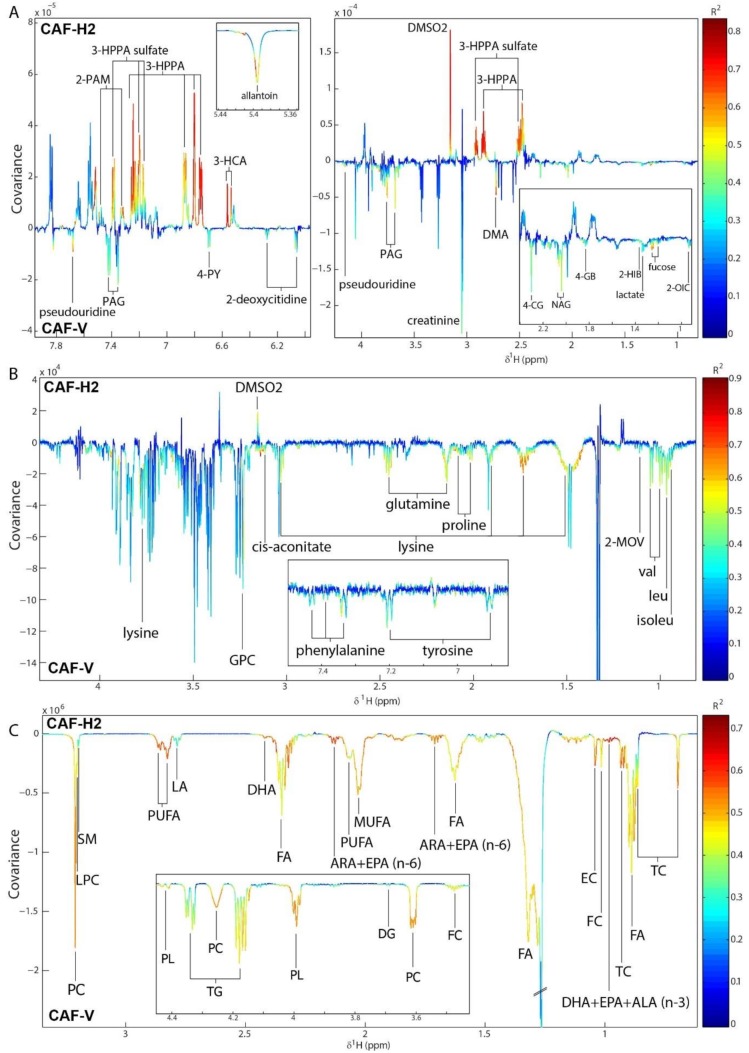
Orthogonal projection to latent structures-discriminant analysis (OPLS-DA) coefficients plot comparing the (**A**) urine metabolic profiles, (**B**) the serum aqueous metabolic profiles, and (**C**) the serum lipid metabolic profiles of CAF-fed rats supplemented with either the vehicle (V) or hesperidin at 100 mg/kg (H2). The colour scale represents the significance of the correlation for each metabolite to the class membership with red indicating strong significance and blue indicating weak significance. 2-HIB, 2-hydroxyisobutyrate, 2-MOV; 3-methyl-2-oxovalerate; 2-OIC; 2-oxoisocaproate; 2-PAM, 2-phenylacetamide; 3-HCA, 3-hydroxycinnamic acid; 3-HPPA, 3-hydroxhyphenylpropionic acid; 4-CG, 4-cresol glucuronide; 4-GB, 4-guanidinobutanoate; ALA, α-linolenic acid; DG, diglycerides; ARA, arachidonic acid; DHA, docosahexaenoic acid; DMA, dimethylamine; DMSO2; dimethylsulfone; EC, esterified cholesterol; EPA, eicosapentaenoic acid; FA, fatty acids; FC; free cholesterol; GPC; glycerophosphocholine; LA, linoleic acid; LPC, lysophosphatidylcholines; MUFA, monounsaturated fatty acids; NAG, *N*-acetylglycoproteins; PAG, phenylacetylglycine; PC, phosphatidylcholines; PL, phospholipids; PUFA, polyunsaturated fatty acids; SM, sphingomyelins; TC, total cholesterol, TG, triglycerides.

**Figure 5:**
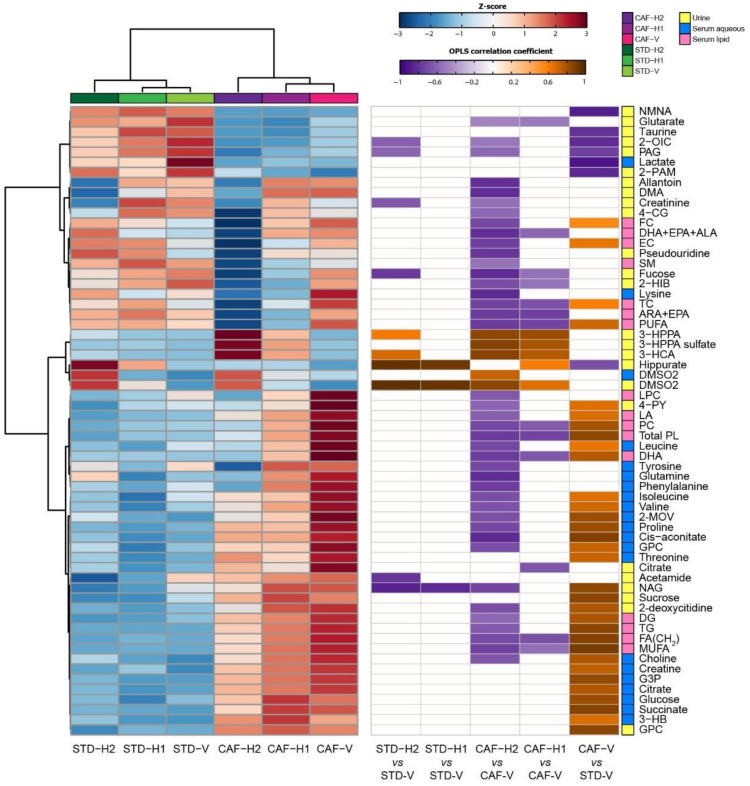
Summary of the diet- and hesperidin-induced metabolic alterations identified by the OPLS-DA models. (Left) Dendrogram and metabolite heatmap displaying the group averages. A metabolite z-transformation was performed on the intensity of each metabolite across samples. Metabolites and groups were clustered by Euclidean distance and Ward linkage hierarchical clustering. (Right) Significant OPLS-DA correlation coefficients associated with the indicated pairwise models. 2-HIB, 2-hydroxyisobutyrate, 2-MOV; 3-methyl-2-oxovalerate; 2-OIC; 2-oxoisocaproate; 2-PAM, 2-phenylacetamide; 3-HCA, 3-hydroxycinnamic acid; 3-HPPA, 3-hydroxhyphenylpropionic acid; 4-CG, 4-cresol glucuronide; 4-GB, 4-guanidinobutanoate; ALA, α-linolenic acid; DG, diglycerides; ARA, arachidonic acid; DHA, docosahexaenoic acid; DMA, dimethylamine; DMSO2; dimethylsulfone; EC, esterified cholesterol; EPA, eicosapentaenoic acid; FA, fatty acids; FC; free cholesterol; GPC; glycerophosphocholine; LA, linoleic acid; LPC, lysophosphatidylcholines; MUFA, monounsaturated fatty acids; NAG, *N*-acetylglycoproteins; PAG, phenylacetylglycine; PC, phosphatidylcholines; PL, phospholipids; PUFA, polyunsaturated fatty acids; SM, sphingomyelins; TC, total cholesterol, TG, triglycerides.

In CAF-fed rats, hesperidin supplementation induced significant alterations in both the urinary and serum metabolic profiles (Table S1). Similar to STD-H1 rats, CAF-H1 rats excreted higher urinary amounts of hippurate, DMSO2, 3-HPPA, and 3-HCA than CAF-V rats. They also excreted higher amounts of 3-HPPA sulfate, but lower amounts of dimethylamine (DMA), fucose, 2-oxoisovalerate (2-OIV), 2-hydroxyisobutyrate (2-HIB), and 4-guanidinobutaonic acid (4-GB) as compared to CAF-V rats (Figures S5 and S6). These metabolic alterations were enhanced after supplementation with H2. Hence, CAF-H2 rats also excreted lower amounts of metabolites related to inflammation (fucose, NAG) and oxidative stress (2-deoxycitidine, pseudouridine, allantoin), uremic toxins (4-cresol glucuronide [4-CG], DMA), guanidino compounds (creatinine, 4-GB), PAG, and 4-PY (Figure 4). Supplementation with H2 also had stronger influence on the serum metabolic profiles. While H1 supplementation only altered the serum lipid profile, H2 supplementation modulated both the lipid and aqueous serum profiles (Table S1). H2 supplementation in CAF rats reverted some of the metabolic alterations observed between CAF and STD rats. Therefore, CAF-H2 rats had lower levels of BCAA, 2-MOV, proline, GPC, TC, FC, EC, FFA, DG, TG, PL, PC, MUFA, and PUFA (Figure 4), all of the metabolites that were increased in CAF rats when compared to STD rats. CAF-H2 rats also had lower levels of other amino acids, such as lysine, glutamine, proline, phenylalanine, and tyrosine, as well as lysophosphocholines (LPC) and sphingomyelins (SM) (Figure 4). A summary of the diet- and hesperidin-induced metabolic alterations identified by pairwise OPLS-DA models is shown in Figure 5.

### 3.8. Integration between Metabolites and the Gut Microbiome

Significant serum and urine metabolites that contribute to the separation between groups were correlated with bacterial families previously [21] identified to change following H2 supplementation in CAF-fed rats (*Bacteroidaceae* and *Ruminococcaceae*). A positive correlation was observed between *Bacteroidaceae* and urinary bacterial-related metabolites, such as 3-HCA, 3-HPPA, and DMSO2 (Figure S7). A strong negative correlation was also observed between this bacterial family and cis-aconitate. No significant correlations were found between metabolites and *Ruminococcaceae*.

## 4. Discussion

The MetS is highly prevalent in Western societies. However, both MetS prevalence and incidence are rapidly increasing in developing countries due to globalization and the widespread of western diet [1]. Therefore, it has become a major global public health problem and a profound burden for national health care systems. Importantly, hesperidin has emerged as a promising therapeutic agent for the treatment of the MetS, due to its wide range of biological properties and the fact that it constitutes 90% of the flavanones in oranges, the most important fruit tree crop in the world [9,10]. Here, we coupled exhaustive biochemical measurements and non-targeted urine and serum metabolomics with multivariate statistical analysis to explore the underlying mechanisms of the effects of hesperidin on rats that were fed a CAF diet. We showed that hesperidin supplementation in CAF-fed animals improved dyslipidaemia and decreased FFA levels. In addition, the highest hesperidin dose also improved insulin sensitivity, hypertension, and markers of arterial stiffness and inflammation. Metabolomics analyses revealed that these changes were accompanied by decreased levels of several serum amino acids, decreased excretion of oxidative stress- and inflammation-related metabolites, and a significant improvement of the lipidomic profile.

A core clinical features of MetS is central obesity, as it usually precedes the emergence of other MetS risk factors [2,3]. As expected, CAF feeding resulted in increased body weight, fat mass, and adiposity index when compared to a STD diet. Obesity also plays a main role in the development of oxidative stress and low-grade inflammation that are associated with the MetS [33]. Consistently, CAF-fed rats displayed higher levels of MCP-1, a molecule that is widely recognized as the major component of chronic inflammation, when compared to STD-fed animals. In addition, metabolomics analyses revealed that the former animals also had higher levels of metabolites that were related to inflammation and oxidative stress. Interestingly, one of the main characteristics of polyphenols is their anti-inflammatory effect that is associated with their antioxidant capacity and the enhancement of the levels of antioxidant enzymes [34]. Consequently, hesperidin supplementation improved the inflammatory and oxidative status of CAF-fed rats, as evidenced by the decreased excretion of NAG, fucose, 2-deoxycitidine, pseudouridine, and allantoin. A protective role of fucose has been suggested in gut-centered and systemic inflammation [35], whereas NAG have recently emerged as useful biomarkers of systemic acute and chronic inflammation [36]. Pseudouridine and 2-deoxycitidine have been used as biomarkers of oxidative stress of DNA and RNA, respectively [37]. Allantoin, which is the end product of purine catabolism, has also been associated with inflammation and oxidative stress. Humans lack the enzyme uricase that converts uric acid to allantoin, so that uric acid is the final compound of purine catabolism [38]. Importantly, several studies have reported a tight relationship between uric acid and MetS [2].

Obesity-related oxidative stress and low-grade inflammation both seem to play a pivotal role in the pathogenesis of IR [39,40], which is the other central clinical feature of MetS. FFA have also been shown to play a substantial role in the onset of IR [2]. In agreement with these findings, a CAF feeding leads to impairment in glucose and insulin homeostasis and increased levels of FFA, which resulted in the development of IR and decreased insulin sensitivity. Therefore, it is likely that the improvement in the oxidative and inflammatory status of CAF-H2 rats might have partly contributed to the significant decrease in serum FFA and increased insulin sensitivity compared to CAF-V rats. In line with our results, the treatment of MetS patients with hesperidin caused a trend towards an improvement of IR [12].

Obesity and IR have also been recognized as the leading causes of MetS associated comorbidities, such as hypertension [41,42] or dyslipidaemia [43]. Hypertension is, by far, the most prevalent individual MetS component, being present in approximately 85% of the MetS patients [3]. FFA also seem to play a possible important role in hypertension development in patient with obesity and MetS [41]. In agreement with our results, the antihypertensive effects of hesperidin have been previously described in spontaneously hypertensive rats [44,45] and, recently, in rats that were fed a high-fat, high-sucrose diet [28]. Interestingly, SBP reduction in CAF-H2 rats was accompanied by an increase in insulin sensitivity and a reduction in FFA. Accumulating evidence indicates that both IR and FFA may promote SBP through endothelial dysfunction and arterial stiffness [40,41,46,47,48]. However, hesperidin supplementation had no effect on the serum levels of ICAM-1 and VCAM-1, both being used as surrogate markers of endothelial dysfunction. Despite this lack of agreement, we must take that we did not find differences between STD- and CAF-fed rats in these parameters into account. In fact, these molecules are not specific of endothelial cells, but can be shed from other cells and, thus, reflect the activity of other biological processes. Nonetheless, CAF rats showed higher levels of neuraminidase, an enzyme that is involved in elastogenesis that has recently been associated with arterial stiffness [49], which is now recognized as a major contributor to hypertension in elderly people [50].

Several studies cross-sectional and prospective cohorts studies revealed positive associations of circulating amino acids, especially BCAA, with visceral obesity, IR, hypertension, dyslipidaemia, MetS, and T2D [51]. In particular, leucine has recently been shown to increase the arterial pressure through the activation of mTORC1signalling [52]. Accordingly, higher levels of BCAA and their catabolites as compared to STD-fed rats characterized the serum metabolic profiles of CAF-fed rats. Notably, the reduction in SBP in CAF-H2 as compared to CAF-V rats was accompanied by a decrease in the circulating levels of BCAA and 2-MOV, an isoleucine catabolite. Recently, 2-MOV has been identified as the strongest predictor of impaired fasting glucose in two independent human cohorts [53].

However, lysine was the amino acid that showed the strongest decrease after hesperidin supplementation in CAF-fed rats when compared to the CAF-V. Lysine competes with arginine for cellular uptake and enhances catabolism by activating kidney arginase, thereby decreasing its bioavailability. Importantly, arginine is the precursor of nitric oxide (NO), which plays a pivotal role in vascular homeostasis and vasodilation. In fact, diminished NO bioavailability is a hallmark of endothelial dysfunction [54]. Therefore, it is tempting to speculate that the reduction of lysine levels in CAF-H2 rats could improve NO bioavailability and partly explain the improvement in SBP that was observed in these animals. In fact, supplementation with free amino acids or protein isolate with a high arginine/lysine ratio reduced SBP and angiotensin-I converting enzyme activity and increased plasma nitrate levels when compared to vehicle supplementation in hypertensive rats [55]. Furthermore, we also found decreased levels of glutamine in CAF-H2, which has been shown to be an important precursor for de novo synthesis of arginine [56].

Increased urinary lysine levels have also been associated with SBP in moderately hypertensive volunteers [57]. This occurred in parallel with increased cis-aconitate levels, which we found to be one of the most discriminatory metabolites between CAF-V and STD-V rats. Similarly, patients with pulmonary arterial hypertension had increased cis-aconitate in the lung [58]. Cis-aconitate is formed from citrate by the enzyme aconitase, and iron dependent enzyme. Interestingly, we found that, in CAF-fed rats, cis-aconitate had the strongest negative correlation with the *Bacteroidaceae* family. We have previously shown that CAF-H2 increased the abundance of *Bacteroidaceae* [21] and it has been reported that *Bacteroides spp.* have shown strong heme-dependence. The high iron demand that is associated with increased levels of this bacterial family in CAF-H2 rats could inhibit the activity of the iron-dependent aconitase and explain the reduced serum cis-aconitate levels.

Most patients with MetS also exhibit dyslipidaemia [2,3]. Consistently, CAF-fed rats had higher circulating levels of TC, LDL-C, TG, and FFA when compared to STD-fed animals. Lipidomic analyses corroborated these results and also revealed and increase in DG, PL, and PC after a CAF diet. One of the most consistent effects of hesperidin is their ability to regulate lipid metabolism [11]. It is in agreement with our lipidomic findings, showing a really significant reduction in all lipid classes in CAF-H2 when compared to CAF-V rats. In addition, a reduction in LDL-C after hesperidin supplementation was the only effect that we also found in STD-fed rats.

Finally, hesperidin supplementation resulted in higher urinary excretions of hippurate, 3-HPPA, and 3-HCA in STD- and CAF-fed rats. These metabolites have been previous described as hesperidin catabolites [59]. In fact, most polyphenols (90–95%) are not absorbed in the small intestine, but they are extensively metabolized in the colon by the enzymatic activity of the gut microbiota to a relatively small number of metabolites that can be absorbed into the bloodstream [17]. Accordingly, the urinary levels of 3-HCA and 3-HPPA correlated positively with the *Bacteroidaceae* family. Importantly, phenolic catabolites may be potentially responsible for the systemic effects of polyphenols [16,17]. For example, 3-HPPA showed considerably higher in vitro vasodilatory effect than quercetin or related flavonoids [60]. Notably, it was the most potent among several phenolic catabolites and its supplementation decreased blood pressure in both normotensive and hypertensive rats. In addition, hippurate excretion was lower in SHR when compared to normotensive Wistar Kyoto rats [61].

DMSO2 was another metabolite that was found in higher levels in both serum and urine after hesperidin supplementation. Little is known regarding DMSO2, but a recent study showed an impressive increase in DMSO2 concentrations in obese subjects after several bariatric procedures [62], which have been shown to produce significant improvements in obesity and hypertension, partly due to changes in the microbiota composition [63,64]. Interestingly, DMSO2 may derive from microbial metabolism of methionine [65], thereby reflecting a change in the gut microbiome composition. Consistently, we found a positive correlation between DMSO2 and Bacteroidaceae, both increased after hesperidin supplementation.

## 5. Conclusions

We demonstrated that hesperidin supplementation in rats that were fed a CAF diet ameliorated most of the MetS components. Specifically, hesperidin supplementation produced a significant decrease in the circulating levels of TC, LDL-C, and FFA in CAF-fed rats. The highest hesperidin dose, H2, also improved insulin sensitivity and decreased SBP and markers of arterial stiffness and inflammation. Metabolomics revealed an improvement in the lipidomic profile, a decrease in circulating amino acids, and a lower excretion of metabolites that are related to inflammation and oxidative stress. CAF-H2 animals excreted higher amounts of microbial-derived metabolites, which positively correlated with *Bacteroidaceae* family. Therefore, the present study provides detailed insights into the effects of hesperidin to attenuate the MetS and supports the hypothesis that hesperidin could be a potential prebiotic for designing strategies for functional foods that aim to reduce the risk of CVD. Further research is needed to further elucidate, in more depth, the underlying molecular mechanism as well as their interaction with the gut microbiota.

## Figures and Tables

**Table 1:** Body composition and dietary and biochemical parameters of rats fed with a standard (STD) or a cafeteria (CAF) diet at the 10th week of the study.

Parameters	STD (*n* = 24)	CAF (*n* = 24)	*p*
*Body composition*			
Weight (g)	422.2 ± 5.2	495.1 ± 9.4	<0.001
Weight change (g)	170.0 ± 3.9	245.7 ± 10.3	<0.001
Fat (%)	6.3 ± 0.4	15.5 ± 1.1	<0.001
Lean (%)	88.5 ± 0.5	80.0 ± 1.1	<0.001
*Dietary parameters*			
Cumulative Energy intake (kcal)	681.8 ± 8.1	1411.3 ± 28.3	<0.001
Cumulative Protein (g)	40.9 ± 0.5	36.2 ± 0.8	<0.001
Cumulative Carbohydrates (g)	97.2 ± 1.2	209.6 ± 4.4	<0.001
Cumulative Fat (g)	13.6 ± 0.2	47.1 ± 1.5	<0.001
Cumulative Fibre (g)	7.7 ± 0.1	4.4 ± 0.2	<0.001
Cumulative SFA (g)	2.0 ± 0.0	18.9 ± 0.5	<0.001
Cumulative MUFA (g)	2.9 ± 0.0	18.1 ± 0.7	<0.001
Cumulative PUFA (g)	7.5 ± 0.1	10.2 ± 0.6	<0.001
Cholesterol (g)	- ^a^	0.22 ± 0.05	<0.001
*Biochemical parameters*			
TC (mmol/L)	22.8 ± 0.6	25.4 ± 0.9	0.013
TG (mmol/L)	1.16 ± 0.11	3.13 ± 0.20	<0.001
FFA(mmol/L)	0.61 ± 0.02	0.82 ± 0.04	<0.001
Glucose (mmol/L)	7.75 ± 0.17	9.10 ± 0.20	<0.001
SBP (mmHg)	128.7 ± 1.1	143.6 ± 1.9	<0.001

^a^ The STD diet had no cholesterol.

## Supplementary Materials

The following are available online at https://www.mdpi.com/2076-3921/9/1/79/s1, Figure S1: NMR spectrum of the hesperidin used in the present study, Figure S2: Study design, Figure S3: OPLS-DA models comparing the metabolic profiles of rats fed a STD or a CAF diet and supplemented with the vehicle (V). (A) urine metabolic profiles, (B) serum aqueous metabolic profile, (C) serum lipid metabolic profile, Figure S4: OPLS-DA models comparing the urine metabolic profiles of STD-fed rats supplemented with either the vehicle (V) or hesperidin at dose1 (H1), Figure S5: OPLS-DA models comparing the urine metabolic profiles of STD-fed rats supplemented with either the vehicle (V) or hesperidin at dose2 (H2), Figure S6: OPLS-DA models comparing the metabolic profiles of CAF-fed supplemented with either the vehicle (V) or hesperidin at dose1 (H1). (A) urine metabolic profile, (B) serum lipid metabolic profile, Figure S7: Correlations between significant metabolites after H2 supplementation and *bacteroidaceae* family in CAF-fed rats. Metabolites in yellow, pink and blue represent urine, serum lipidic, and serum aqueous metabolites, Table S1: Models characteristics.
