# Supplementary material for: Metabolomics Elucidates Dose-Dependent Molecular Beneficial Effects of Hesperidin Supplementation in Rats Fed an Obesogenic Diet
Source: Antioxidants (Basel). 2020 Jan 16;9(1):79. doi: 10.3390/antiox9010079 (PMC7023145; doi:10.3390/antiox9010079)

Supplementary Figure 1. NMR spectrum of the hesperidin extract used in the present study. The peaks 2.86-2.90 ppm were used to determine the proportions of 2S and 2R enantiomers.

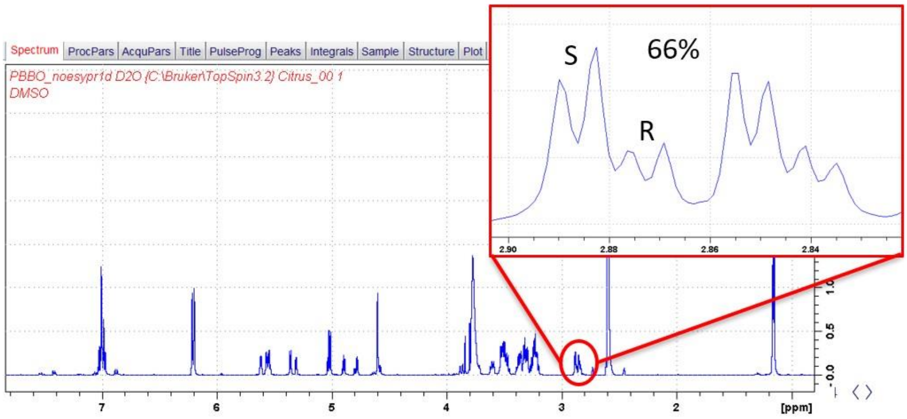

Supplementary Figure 2. Study design. CAF, cafeteria diet; H, hesperidin; STD, standard chow diet; V, vehicle.

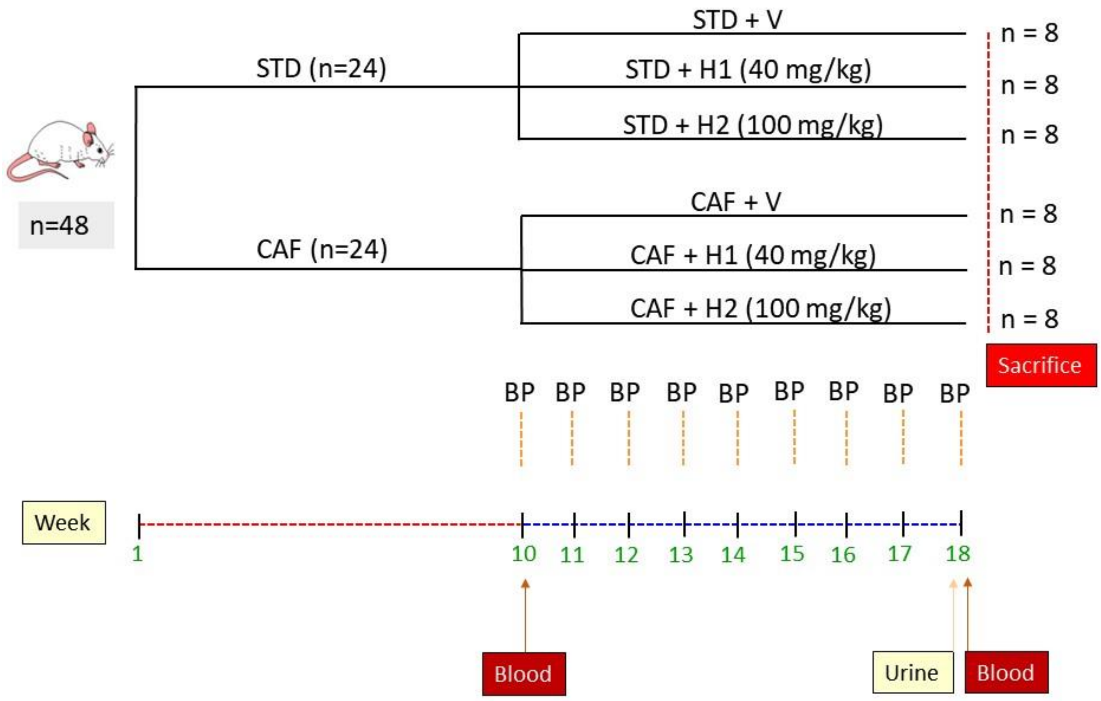

Supplementary Table 1. Models characteristics. Significant models are shown in bold.

Model	Ortho components	R^2Y	Q^2Y	*P*
Urine				
**STD-V vs CAF-V**	**1**	**1.0**	**0.81**	**<0.001**
**STD-V vs STD-H1**	**7**	**1.0**	**0.84**	**<0.001**
**STD-V vs STD-H2**	**1**	**0.98**	**0.87**	**<0.001**
STD-H1 vs STD-H2	8	1.0	0.71	0.30
**CAF-V vs CAF-H1**	**2**	**1.0**	**0.63**	**0.006**
**CAF-V vs CAF-H2**	**7**	**1.0**	**0.93**	**<0.001**
CAF-H1 vs CAF-H2	3	1.0	0.40	0.15
Serum AQ				
**STD-V vs CAF-V**	**2**	**1.0**	**0.58**	**<0.001**
STD-V vs STD-H1	4	1.0	0.54	0.17
STD-V vs STD-H2	4	1.0	0.58	0.21
STD-H1 vs STD-H2	4	1.0	0.49	0.36
CAF-V vs CAF-H1	3	1.0	0.36	0.60
**CAF-V vs CAF-H2**	**2**	**1.0**	**0.62**	**0.017**
CAF-H1 vs CAF-H2	4	1.0	0.82	0.03
Serum LIP				
**STD-V vs CAF-V**	**5**	**1.0**	**0.81**	**<0.001**
STD-V vs STD-H1	0	0.48	-0.34	-
STD-V vs STD-H2	0	0.44	-0.06	-
STD-H1 vs STD-H2	0	0.36	-0.13	-
**CAF-V vs CAF-H1**	**0**	**0.48**	**0.30**	**0.034**
**CAF-V vs CAF-H2**	**0**	**0.60**	**0.51**	**0.005**
CAF-H1 vs CAF-H2	0	0.49	-0.35	-

Supplementary Figure 3. OPLS-DA models comparing the metabolic profiles of rats fed a STD or a CAF diet and supplemented with the vehicle (V). A) urine metabolic profiles, B) serum aqueous metabolic profile, C) serum lipid metabolic profile.

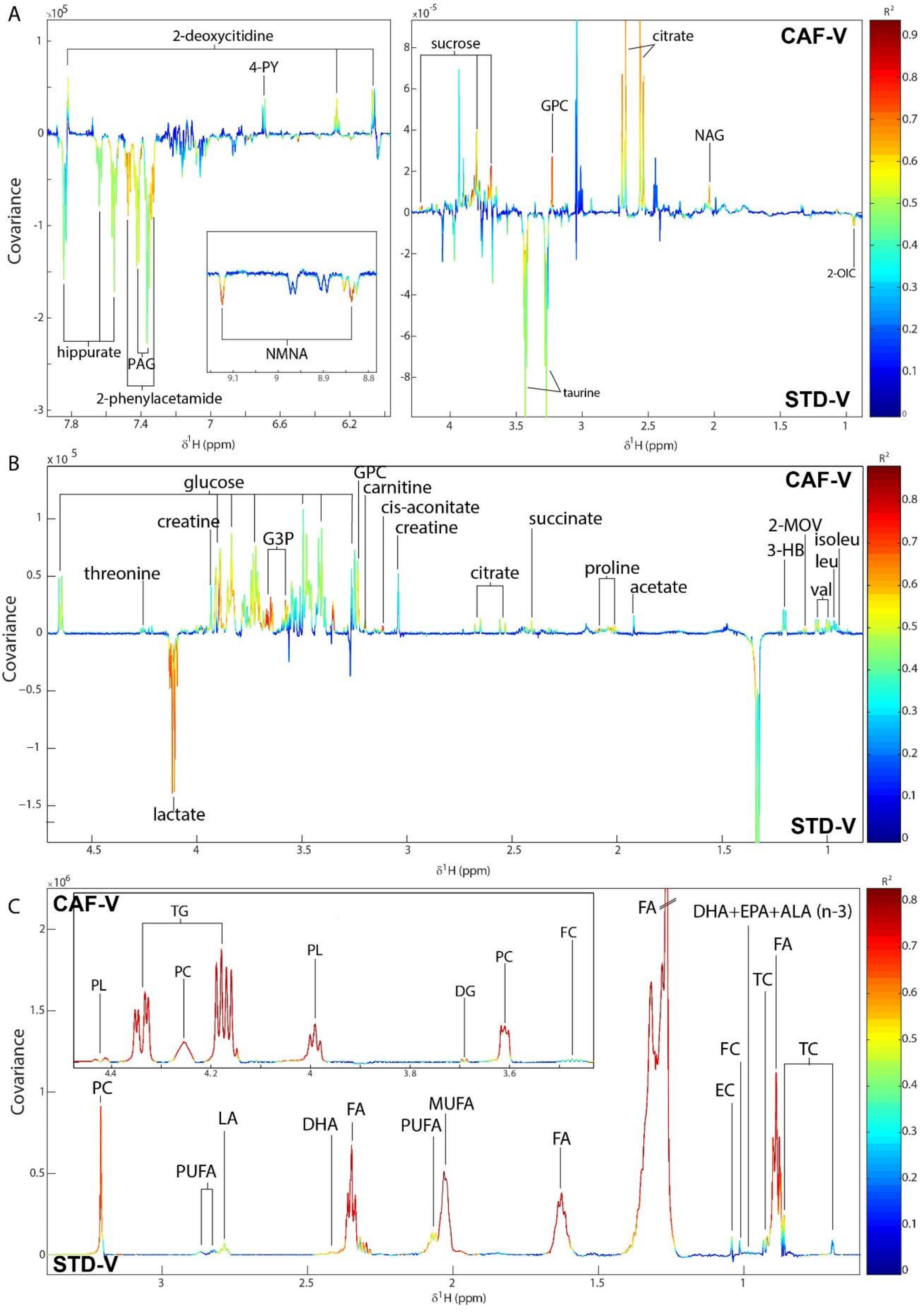

15 **Supplementary Figure 4.** OPLS-DA models comparing the urine metabolic profiles of STD-fed rats supplemented with either the vehicle (V) or hesperidin at dose1 (H1).

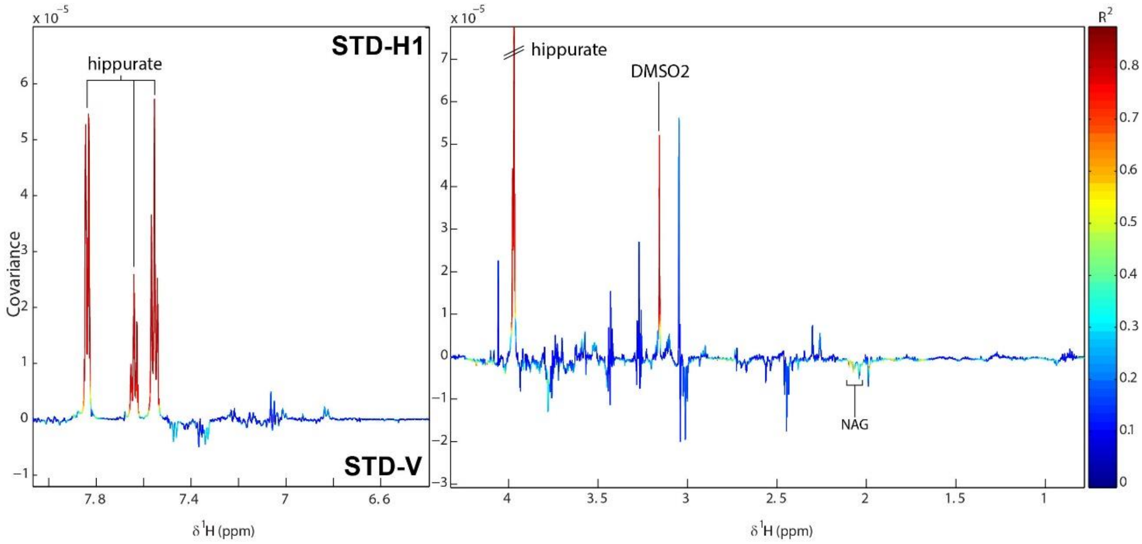

Supplementary Figure 5. OPLS-DA models comparing the urine metabolic profiles of STD-fed rats supplemented with either the vehicle (V) or hesperidin at dose2 (H2).

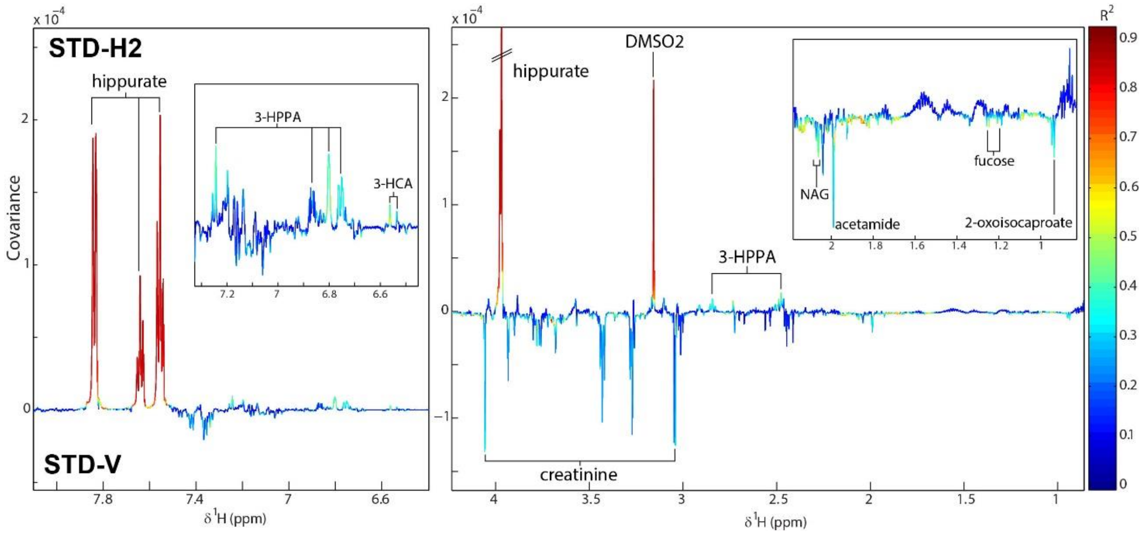

Supplementary Figure 6. OPLS-DA models comparing the metabolic profiles of CAF-fed supplemented with either the vehicle (V) or hesperidin at dose1 (H1). A) urine metabolic profile, B) serum lipid metabolic profile.

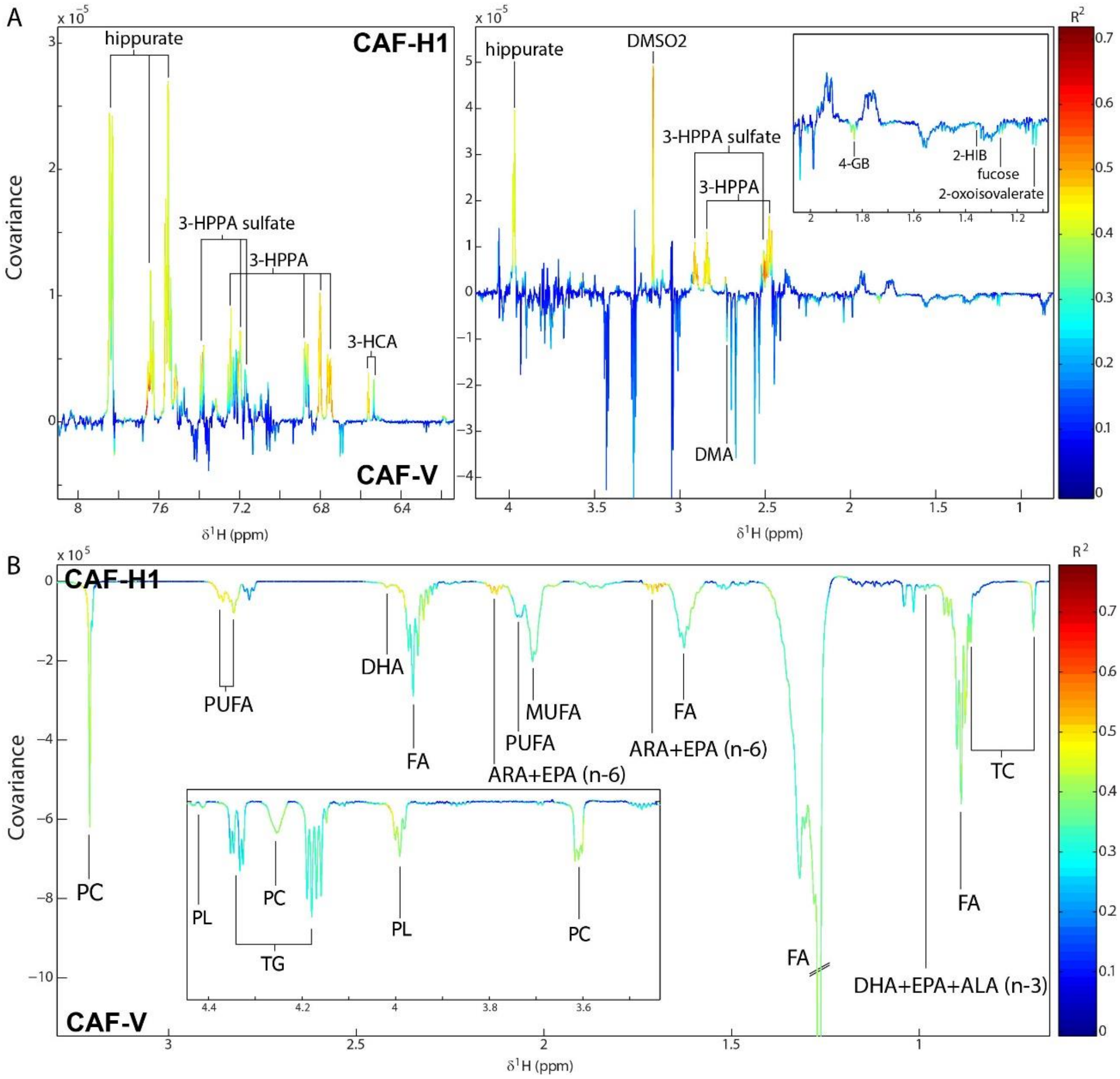

Supplementary Figure 7. Correlations between significant metabolites after H2 supplementation and *bacteroidaceae* family in CAF-fed rats. Metabolites in yellow, pink and blue represent urine, serum lipidic, and serum aqueous metabolites.

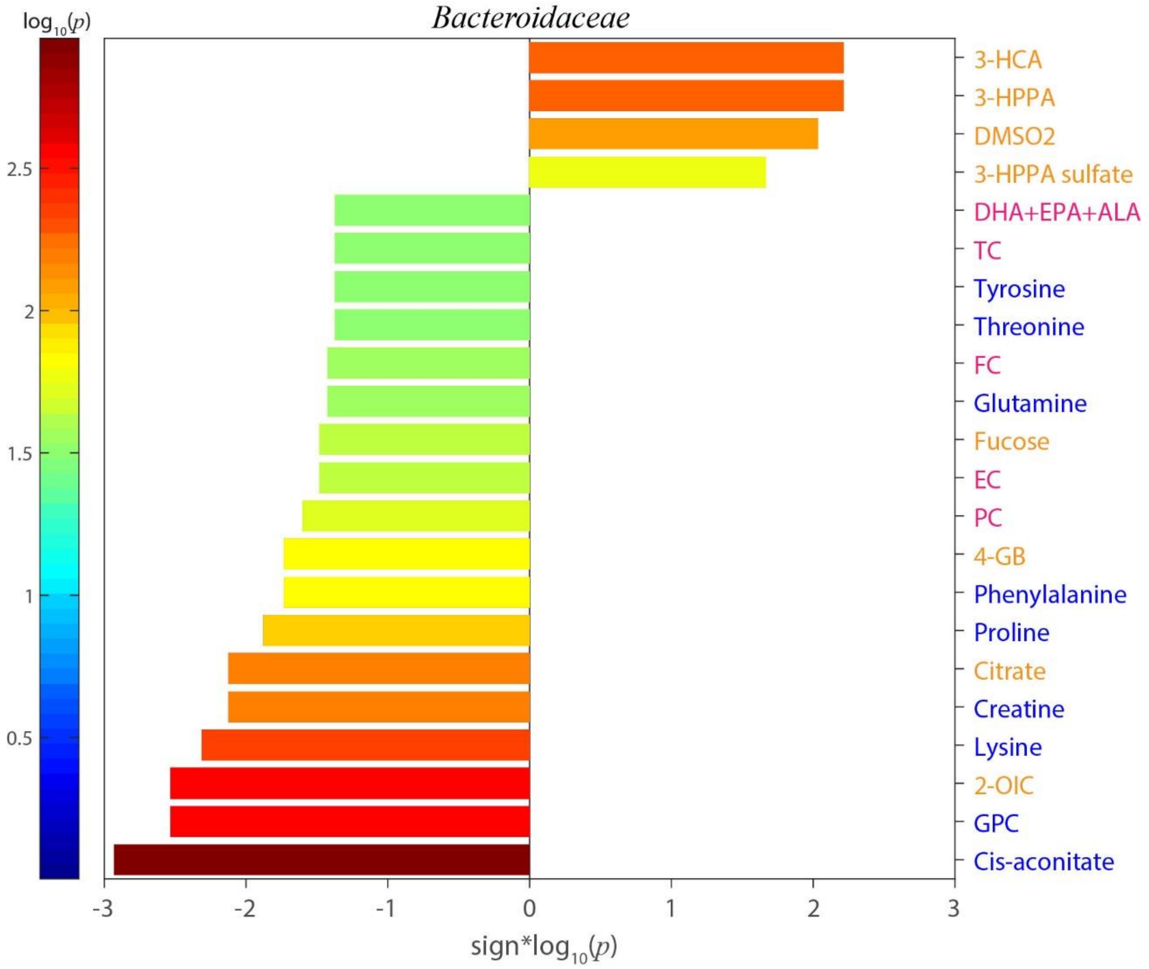

Supplement: Supplementary file 1 [file antioxidants-09-00079-s001.pdf]
